# *Arabidopsis* AL PHD-PRC1 Complexes Promote Seed Germination through H3K4me3-to-H3K27me3 Chromatin State Switch in Repression of Seed Developmental Genes

**DOI:** 10.1371/journal.pgen.1004091

**Published:** 2014-01-23

**Authors:** Anne Marie Molitor, Zhongyuan Bu, Yu Yu, Wen-Hui Shen

**Affiliations:** 1 Institut de Biologie Moléculaire des Plantes du CNRS, Université de Strasbourg, Strasbourg, France; 2 State Key Laboratory of Genetic Engineering, International Associated Laboratory of CNRS-Fudan-HUNAU on Plant Epigenome Research, School of Life Sciences, Fudan University, Shanghai, PR China; University of Edinburgh, United Kingdom

## Abstract

Seed germination and subsequent seedling growth define crucial steps for entry into the plant life cycle. For those events to take place properly, seed developmental genes need to be silenced whereas vegetative growth genes are activated. Chromatin structure is generally known to play crucial roles in gene transcription control. However, the transition between active and repressive chromatin states during seed germination is still poorly characterized and the underlying molecular mechanisms remain largely unknown. Here we identified the *Arabidopsis* PHD-domain H3K4me3-binding ALFIN1-like proteins (ALs) as novel interactors of the Polycomb Repressive Complex 1 (PRC1) core components AtBMI1b and AtRING1a. The interactions were confirmed by diverse *in vitro* and *in vivo* assays and were shown to require the AL6 N-terminus containing PAL domain conserved in the AL family proteins and the AtRING1a C-terminus containing RAWUL domain conserved in animal and plant PRC1 ring-finger proteins (including AtRNIG1a/b and AtBMI1a/b). By T-DNA insertion mutant analysis, we found that simultaneous loss of AL6 and AL7 as well as loss of AtBMI1a and AtBMI1b retards seed germination and causes transcriptional derepression and a delayed chromatin state switch from H3K4me3 to H3K27me3 enrichment of several seed developmental genes (*e.g. ABI3*, *DOG1*, *CRU3*, *CHO1*). We found that AL6 and the PRC1 H3K27me3-reader component LHP1 directly bind at *ABI3* and *DOG1* loci. In light of these data, we propose that AL PHD-PRC1 complexes, built around H3K4me3, lead to a switch from the H3K4me3-associated active to the H3K27me3-associated repressive transcription state of seed developmental genes during seed germination. Our finding of physical interactions between PHD-domain proteins and PRC1 is striking and has important implications for understanding the connection between the two functionally opposite chromatin marks: H3K4me3 in activation and H3K27me3 in repression of gene transcription.

## Author Summary

Seed germination is a key developmental transition during the plant life cycle. During this process, seed developmental genes are progressively turned off to allow proper seedling growth and development. Molecular mechanisms involved in establishing the repression state of seed developmental genes, however, are still poorly known. Here we demonstrated that the *Arabidopsis* PHD-domain H3K4me3-binding ALFIN1-like proteins (ALs) physically interact with the Polycomb Repressive Complex 1 (PRC1) core components AtBMI1b and AtRING1a. Loss of AL6 and AL7 as well as loss of AtBMI1a and AtBMI1b causes transcriptional derepression and a delayed chromatin state switch from H3K4me3 to H3K27me3 enrichment of several seed developmental genes (*e.g. ABI3*, *DOG1*, *CRU3*, *CHO1*). Furthermore, GFP-AL6 and LHP1-myc directly bind at *ABI3* and *DOG1* loci. We propose that AL PHD-PRC1 complexes, built around H3K4me3, lead to a switch from the H3K4me3-associated activation to the H3K27me3-associated repression of seed developmental genes. Our study unravels a striking connection between PHD-domain proteins and PRC1, two types of readers for the functionally opposite chromatin marks. The newly uncovered mechanism is relevant to understand reprogramming of gene activity, which is at the heart of plant growth phase transitions and of cell differentiations in plants and other organisms.

## Introduction

Timely transition from a growth-arrested seed to a growing seedling is a key process during the plant life cycle, which has great importance in plant adaptation to environmental conditions [1]. Seed germination, a developmental process spanning from initial seed hydration to embryonic root emergence, and subsequent seedling growth requires silencing of seed developmental genes, *e.g. ABSCISIC ACID INSENSITIVE 3* (*ABI3*) and *DELAY OF GERMINATION 1* (*DOG1*) in *Arabidopsis thaliana*. ABI3 belongs to the plant-specific B3 domain transcription factor family and regulates the expression of a number of genes involved in various aspects of seed development [2]–[4]. Among those, the 12S seed storage protein encoding genes *CRUCIFERIN1* (*CRU1/CRA1*), *CRU2* and *CRU3*/*CRC* are bound by ABI3 [5], likely representing direct targets of ABI3. Although the precise function of the DOG1 protein remains unknown, *DOG1* is a major quantitative trait locus for seed dormancy and its transcript as well as protein levels are tightly regulated during seed development [6]–[8]. Among other regulatory genes, the AP2 family transcription factor gene *CHOTTO1*/*AINTEGUMENTA-LIKE5* (*CHO1*/*AIL5*) and the antioxidant gene *CYSTEINE PEROXIREDOXIN 1 (PER1)* also show specific seed expression patterns and regulate *Arabidopsis* seed germination under certain laboratory conditions, such as nitrate, salt and glucose treatments [9], [10].

Recent studies implicate the requirement of the repressive histone mark H3K27me3 in silencing of seed developmental genes during seedling growth [11]–[15]. The H3K27me3 mark is established by the Polycomb Repressive Complex 2 (PRC2), which is conserved in animals and plants [16]. *Arabidopsis* PRC2 mutant seedlings show substantially reduced levels of H3K27me3 and ectopic expression of seed developmental genes, including *ABI3*, *DOG1*, *CRU1*, *CRU3*, and *CHO1* [12]. Loss of function of the *Arabidopsis* ATP-dependent chromatin remodeler PICKLE also affects H3K27me3 deposition and *ABI3* repression [11], [15], [17]. It is generally considered that H3K27me3 provides a docking site for the PRC1 complex, which subsequently conducts H2A monoubiquitination and establishes a repressive chromatin configuration [18]. PRC1 components diverge considerably between animals and plants, and the most conserved components are the ring-finger proteins RING1 and BMI1 (reviewed in Molitor and Shen [19]). The *Arabidopsis* double mutants *Atring1a Atring1b* and *Atbmi1a Atbmi1b* display pleiotropic phenotypes, including ectopic embryonic callus formation and *ABI3* derepression in seedlings [20]–[23]. In spite of the importance of PRC2 and PRC1 in silencing, however, how seed developmental genes timely switch from activation to a repression chromatin state is not understood.

In this study we identify the ALFIN1-like (AL) PHD-domain proteins, which bind H3K4me2/me3 [24], as novel partners of AtRING1 and AtBMI1. We provide evidence supporting a role of AL PHD-PRC1 complexes during the chromatin state switch from H3K4me3-associated transcriptional activation to H3K27me3-associated transcriptional repression of seed developmental genes, *i.e. ABI3*, *DOG1*, *CRU1*, *CRU3*, *CHO1* and *PER1*, which is necessary for proper seed germination and early seedling growth.

## Results

### Identification of AL proteins as PRC1 interactors

**Figure 1:**
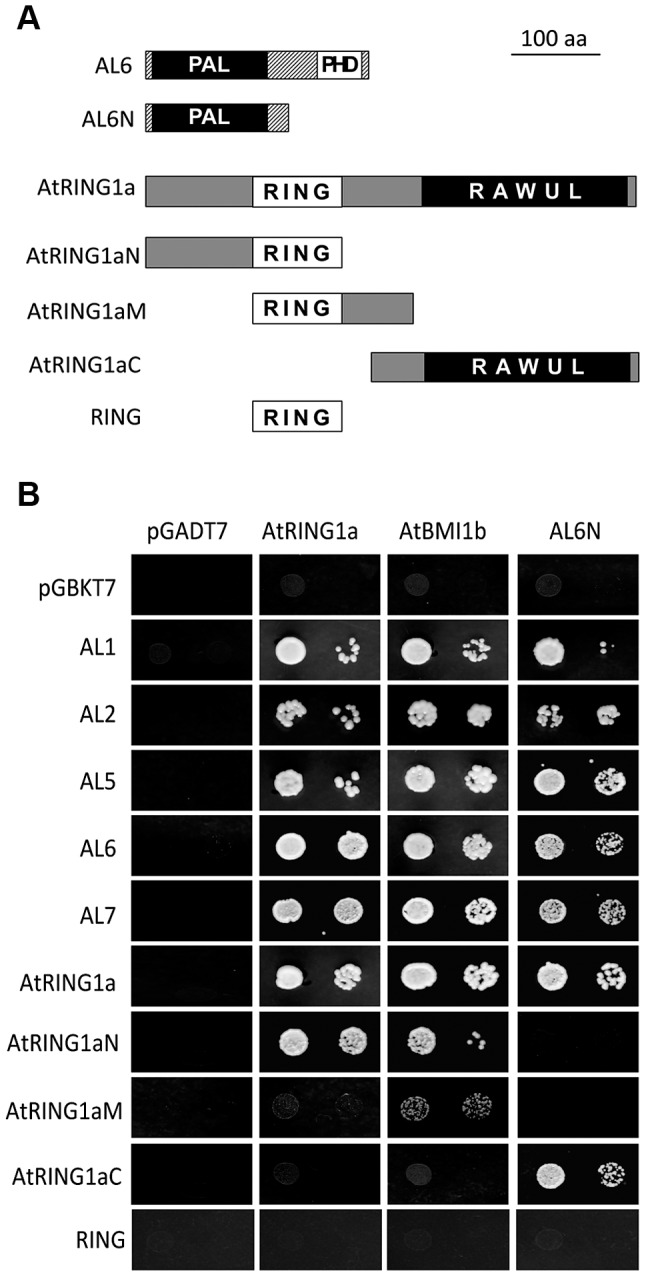
Interactions of ALs and PRC1 ring-finger proteins in yeast two-hybrid assay. (A) Schematic representation of full-length and truncated AtRING1a and AL6 proteins. The conserved domains PAL, PHD, RING and RAWUL are indicated. (B) Yeast two-hybrid assays. Yeast cultures co-expressing the indicated protein combinations from pGADT7 and pGBKT7 were plated as a 1∶10 dilution from left to right onto SD-LTA selective media. Growth of yeast cells indicates positive protein-protein interaction.

During a yeast two-hybrid screen using AtRING1a as bait, we isolated from an *Arabidopsis* seedling cDNA library three positive clones corresponding to *AL6*. *AL6* encodes a small protein of 256 amino acids, which contains a PHD domain at the C-terminus (Figure 1A and Figure S1). The AL group proteins are found in the green lineage including algae and plants but not in fungi and animals. The *Arabidopsis* genome contains seven homologs denoted AL1 to AL7 ([24]; Figure S1). In the yeast two-hybrid test, AL1, AL2, AL5, AL6 and AL7 each interact with AtRING1a and AtBMI1b. Deletion analysis revealed that the N-terminal region of AL6 excluding the PHD domain could bind the C-terminal region of AtRING1a excluding the RING domain (Figure 1B). It is worth noting that the N-terminal region of AL6 could also interact with ALs, suggesting that ALs might form homo- and/or hetero-dimers. The N-terminus of AL6 contains a domain of so far unknown function (DUF3594), which is specifically conserved in the AL group proteins and thus likely represents a novel protein-binding domain (Figure S1). We named this domain PAL for PHD-associated AL domain. The C-terminus of AtRING1a contains the RAWUL domain, an ubiquitin-like domain conserved in animal and plant PRC1 ring-finger proteins including AtRING1a, AtRING1b, AtBMI1a, and AtBMI1b [25]. Our data suggest that PAL and RAWUL may form a protein-protein interaction module. In agreement with previous observations [20]–[22], AtRING1a could interact with itself and AtBMI1b, and this interaction was shown here to occur *via* the N-terminal region containing the RING domain (Figure 1A and 1B).

**Figure 2:**
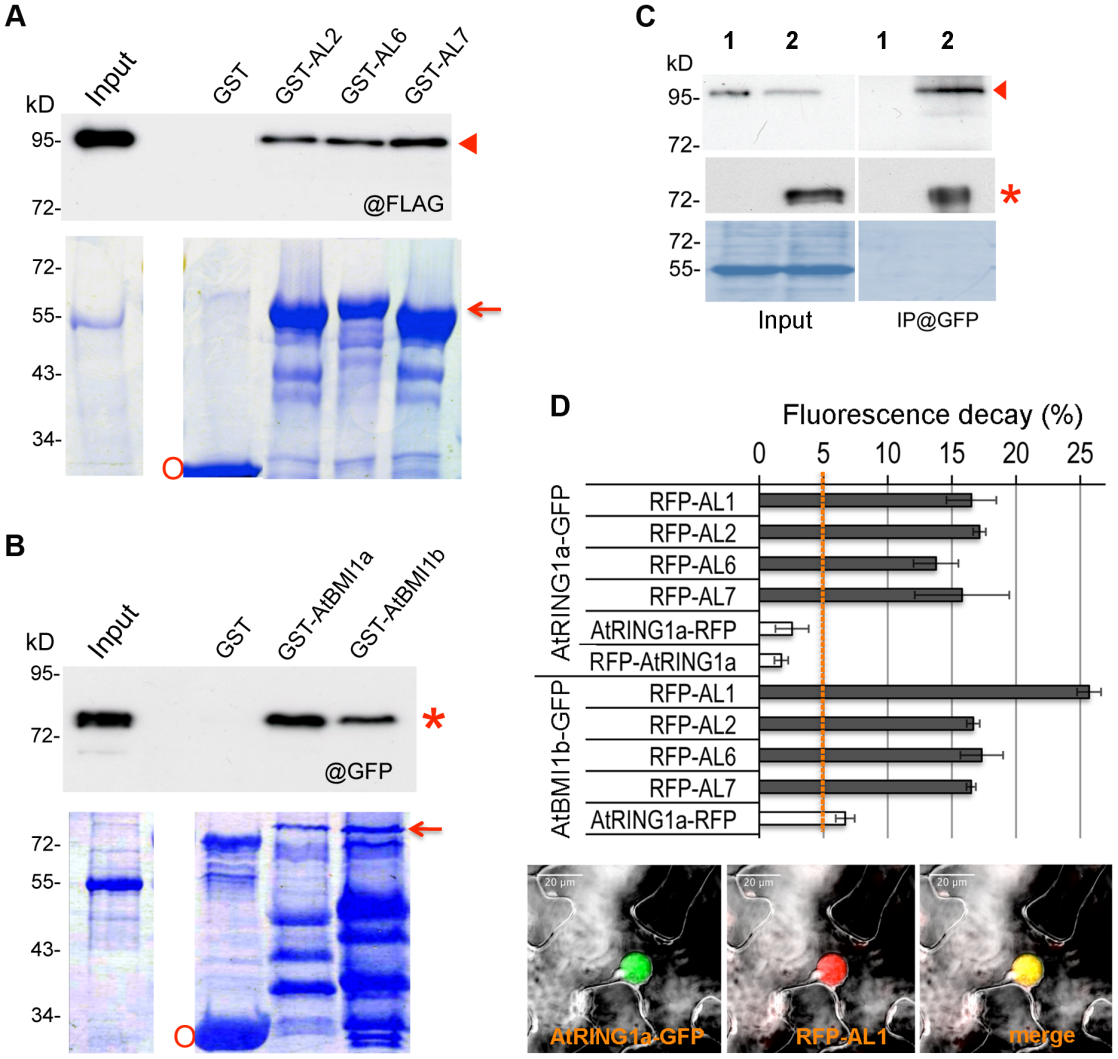
ALs physically interact with PRC1 ring-finger proteins in both *in vitro* and *in vivo* assays. (A) Pulldown assay. Agarose beads coated with GST, GST-AL2, GST-AL6 or GST-AL7 were incubated with an equal aliquot of total protein extracts of *Arabidopsis* plants expressing *FLAG-AtRING1a*. The pulldown fractions and inputs were analyzed by Western blot using antibodies against FLAG (@FLAG, top panel). Coomassie staining is shown as loading control (bottom panel). The positions of FLAG-AtRING1a, GST-AL and GST are indicated by arrowhead, arrow and circle, respectively. (B) Pulldown assay. Agarose beads coated with GST, GST-AtBMI1a or GST-AtBMI1b were incubated with an equal aliquot of total protein extracts of *Arabidopsis* plants expressing *GFP-AL6*. The pulldown fractions and inputs were analyzed by Western blot using antibodies against GFP (@GFP, top panel). Coomassie staining is shown as loading control (bottom panel). The positions of GFP-AL6, GST-AtBMI1 and GST are indicated by star, arrow and circle, respectively. (C) Co-IP detection of AtRING1a and AL6 interaction *in planta.* Total protein extracts from plants expressing *FLAG-AtRING1a* alone (lane 1) or both *FLAG-AtRING1a* and *GFP-AL6* (lane 2) were immunoprecipitated with a polyclonal anti-GFP antibody, and the resulting fractions were analyzed by Western blot using anti-FLAG (top panel) or HRP-conjugated anti-GFP (middle panel) monoclonal antibodies. Coomassie staining (bottom panel) is shown as loading quality control. Arrowhead and star indicate FLAG-AtRING1a and GFP-AL6 positions, respectively. (D) FLIM detection of AtRING1a-GFP or AtBMI1b-GFP interaction with RFP-ALs *in planta*. GFP- and RFP-tagged proteins as indicated were transiently co-expressed in *Nicotiana benthamiana* leaves. The fluorescence lifetime of GFP fusion proteins was recorded two days post infiltration. Data represent average GFP fluorescence lifetime decay ± SD of three biological replicates, each recording over 30 nuclei. Values above 5% indicate positive protein-protein interactions. The bottom image panels show co-localization of AtRING1a-GFP and RFP-AL1 in the nucleus of a leaf epidermal cell.

Next, we verified interactions between ALs and PRC1 ring-finger proteins by several independent techniques. Using *in vitro* pulldown assays, we observed that GST-fused AL2, AL6 or AL7 but not GST alone could pulldown FLAG-AtRING1a from total protein extracts of transgenic plants (Figure 2A). Similarly, GST-AtBMI1a or GST-AtBMI1b could pulldown GFP-AL6 (Figure 2B). In co-immunoprecipitation (CoIP) experiments, FLAG-AtRING1a was detected in the GFP-AL6 immunoprecipitated fraction from transgenic plants expressing *FLAG-AtRING1a* and *GFP-AL6* (Figure 2C). Finally, using Fluorescence Lifetime Imaging Microscopy (FLIM) analysis, we detected interaction of AtRING1a-GFP with RFP-AL1, RFP-AL2, RFP-AL6 or RFP-AL7 but not with AtRING1a-RFP or RFP-AtRING1a, as well as interaction of AtBMI1b-GFP with RFP-AL1, RFP-AL2, RFP-AL6, RFP-AL7 or AtRING1a-RFP (Figure 2D).

Collectively, these data firmly establish that AL proteins are interactors of the PRC1 ring-finger component proteins, AtRING1 and AtBMI1.

### The *al6 al7* and *Atbmi1a Atbmi1b* mutants display germination hypersensitivity to osmotic stress

**Figure 3:**
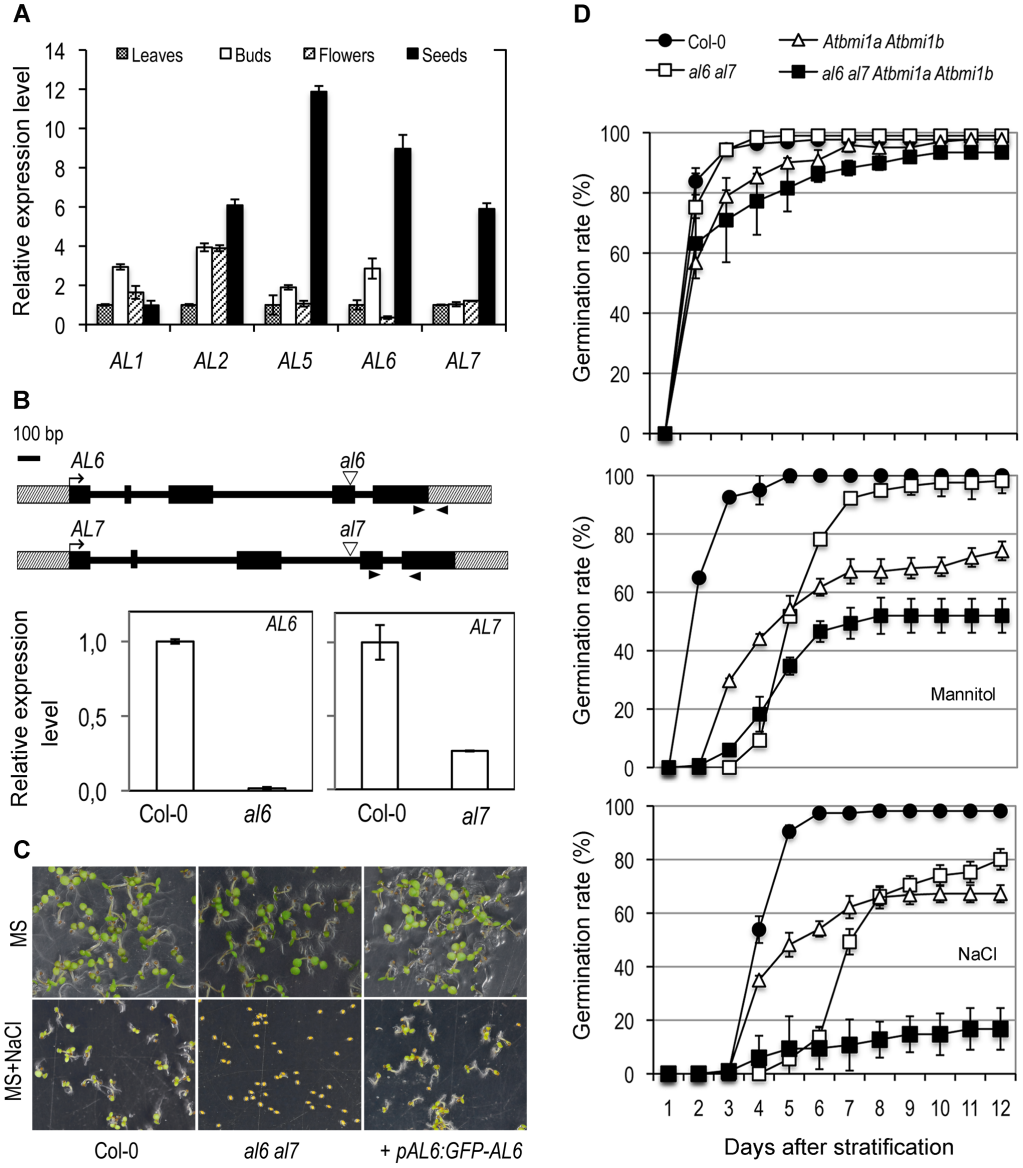
Functional characterization of *AL* genes. (A) Tissue-specificity of *AL* gene expression. Relative expression levels of *AL1*, *AL2*, *AL5*, *AL6*, and *AL7* were determined by quantitative RT-PCR in different plant organs. Leaves: rosette leaves from 4-week-old plants; Buds: floral buds before anthesis; Flowers: flowers at anthesis; Seeds: dry seeds. Data represent means ± SD of three biological replicates. (B) *AL6* and *AL7* genomic structure and T-DNA insertion mutants. Genes are schematically represented by black boxes for exons, black lines for introns and dashed boxes for untranslated regions. Triangles indicate T-DNA insertion sites and arrowheads indicate RT-PCR primer positions. Relative expression levels of *AL6* and *AL7* in Col-0 and in *al6* and *al7* mutants are shown as means ± SD of three biological replicates. (C) Representative seed germination images of Col-0, *al6 al7* double mutant, and the double mutant complemented by the *AL6* promoter driving *GFP-AL6* fusion gene (*+pAL6:GFP-AL6*). Images were taken five days after stratification from plates containing MS media or MS supplemented with 100 mM NaCl (MS+NaCl). (D) Germination rate of Col-0, double mutants *al6 al7* and *Atbmi1a Atbmi1b*, and the quadruple mutant *al6 al7 Atbmi1a Atbmi1b* plated on MS (top graph), MS supplemented with 200 mM mannitol (middle graph) or with 100 mM NaCl (bottom graph). Data represent average germination percentages ± SD of three biological replicates, each >60 seeds, observed daily for 12 days after stratification.

*AL* gene expression was detected ubiquitously but at varied levels in different plant organs (Figure 3A). Hereinafter we focused on the functional characterization of *AL6* and *AL7*, two genes preferentially expressed in seeds and encoding two proteins with 84% identity at the amino acid sequence level, which are grouped in a separate clade from the other AL proteins according to phylogenetic analysis (Figure S2). T-DNA insertion mutants, *al6* and *al7*, were obtained and shown to display knockdown of *AL6* and *AL7*, respectively (Figure 3B). Under standard growth conditions, the *al6* and *al7* mutants as well as the *al6 al7* double mutant showed a normal growth phenotype. However, under osmotic treatments with salt or mannitol, the *al6 al7* double mutant but not the single mutants showed a delay of seed germination compared to the wild-type Col-0 (Figure 3C and 3D). This mutant phenotype could be rescued by transformation with *pAL6:GFP-AL6* (Figure 3C), indicating that *AL6* and *AL7* act redundantly and knockdown of both *AL6* and *AL7* function has caused the mutant phenotype.

The *Atbmi1a Atbmi1b* mutant also displayed a delay in seed germination in our assays (Figure 3D). Interestingly, compared to that of *al6 al7*, the exponential phase of the *Atbmi1a Atbmi1b* germination rate curve started earlier after stratification, but reaching a comparatively lower maximum percentage value. This observation suggests that *AL6/7* may be involved primarily in initiation and *AtBMI1a/b* in maintenance of the germination process. We also obtained the quadruple mutant *al6 al7 Atbmi1a Atbmi1b* and showed that it is drastically impaired in both germination initiation time and maximum percentage of germination rate (Figure 3D). The enhanced germination defects observed in the quadruple mutant further suggest that other ALs and PRC1 ring-finger proteins may also participate in germination regulation.

### Seed developmental genes are derepressed in *al6 al7* and *Atbmi1a Atbmi1b* mutants

**Figure 4:**
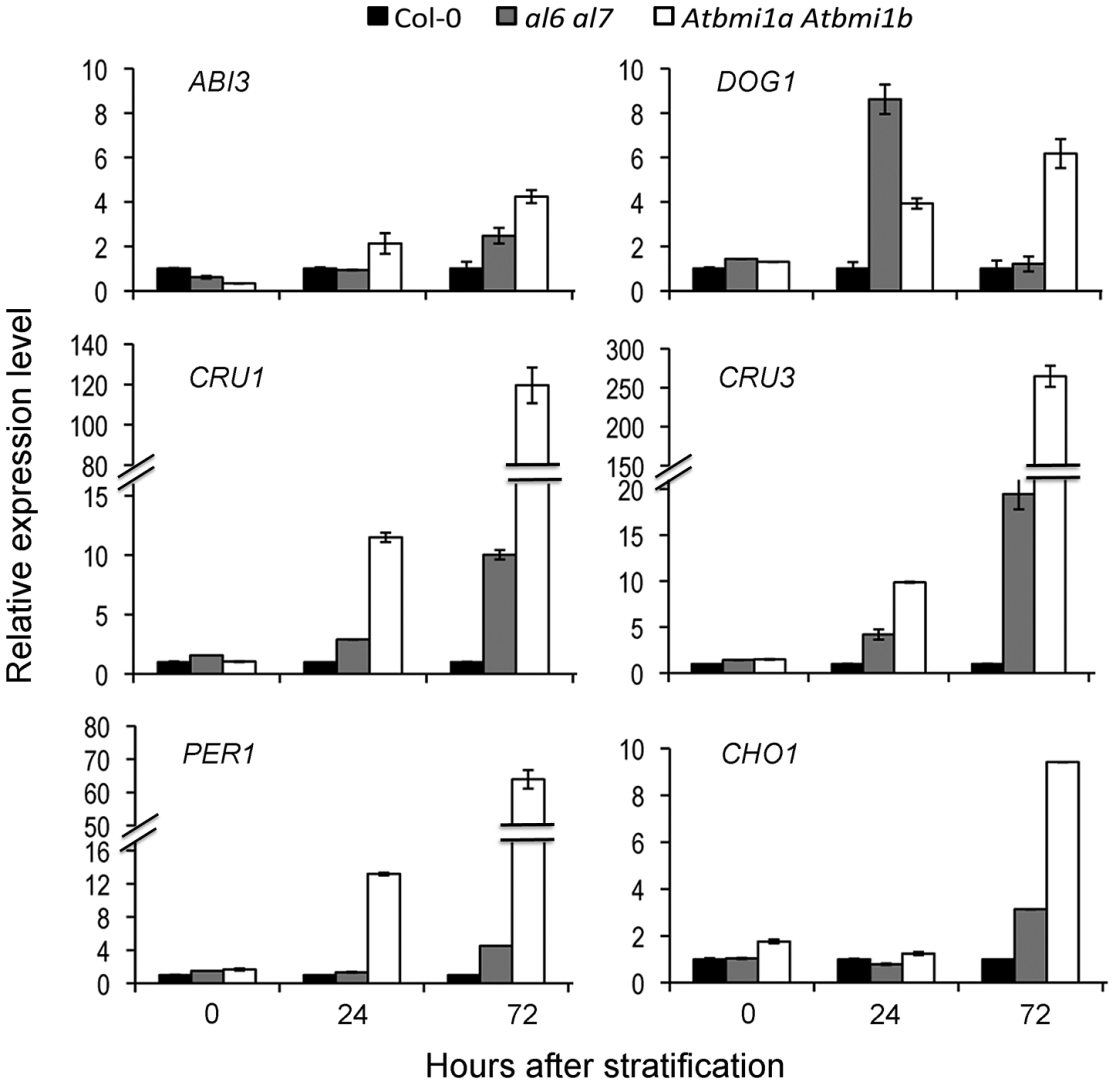
Relative expression levels of seed developmental genes in Col-0, *al6 al7* and *Atbmi1a Atbmi1b*. Relative expression levels of *ABI3*, *DOG1*, *CRU1*, *CRU3*, *PER1* and *CHO1* were analyzed by quantitative RT-PCR using seeds/seedlings at 0, 24 and 72 hours after stratification. Data represent means ± SD of three biological replicates.

As expected, the six examined seed developmental genes *ABI3*, *DOG1*, *CRU1*, *CRU3*, *CHO1* and *PER1* displayed transcriptional repression during the 72 hours after stratification (HAS; Figure S3A and S3B). Notably, *AL5*, *AL6* and *AL7* genes, but not *AL1* and *AL2* genes, also showed transcription repression during seed germination (Figure S3C). In line with the germination delay, salt treatment inhibited the repression of seed developmental genes as well as that of *AL5*, *AL6* and *AL7* albeit to a less extent (Figure S3). Meanwhile, it is worth to note that *AL1*, *AL2*, *AL6* and *AL7* are well expressed after seed germination and along different plant developmental stages (Figure S4). Next, we investigated expression levels of the six seed developmental genes in the *al6 al7* and *Atbmi1a Atbmi1b* mutants. Because the salt treatment drastically varied the germination time of Col-0, *al6 al7* and *Atbmi1a Atbmi1b*, to minimize secondary effects we choose to perform molecular analyses using seeds/seedlings germinated on normal media where wild-type and mutants only exhibit very little differences (Figure 3D). As shown in Figure 4, all six seed developmental genes showed higher expression levels in the *al6 al7* and *Atbmi1a Atbmi1b* mutants as compared to Col-0 at 72 HAS, with some genes (*e.g. DOG1*, *CRU1*, *CRU3*, *PER1*) also elevated earlier at 24 HAS. These data demonstrate that both *AL6/7* and *AtBMI1a/b* are involved in repression of seed developmental genes during germination and early seedling growth.

### Timely switch from H3K4me3- to H3K27me3-marked chromatin state of seed developmental genes is impaired in *al6 al7* and *Atbmi1a Atbmi1b* mutants during seed germination

**Figure 5:**
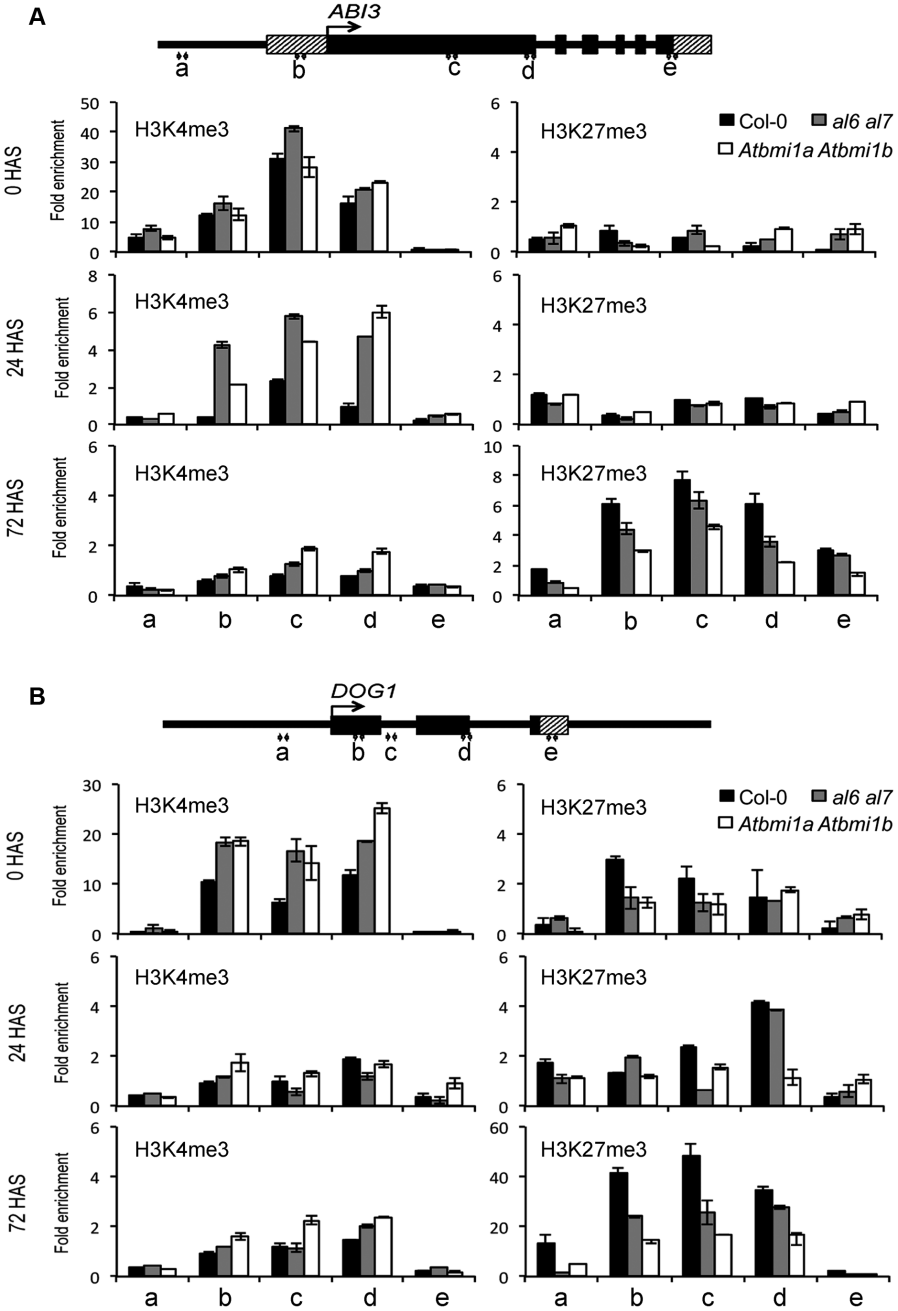
Relative enrichments of H3K4me3 and H3K27me3 at seed developmental genes in Col-0, *al6 al7* and *Atbmi1a Atbmi1b*. H3K4me3 and H3K27me3 levels were analyzed by ChIP at five regions (a to e) of *ABI3* (A) and *DOG1* (B). Gene structures are schematically represented by black boxes for exons, black lines for introns and promoters, and dashed boxes for untranslated regions. Seeds/seedlings at 0, 24 and 72 hours after stratification (HAS) were analyzed. Values were normalized to internal controls (relative to input and to *TUB2*). Data represent means ± SD of three biological replicates.

To investigate the mechanism of seed gene repression, we performed chromatin immunoprecipitation (ChIP) analysis on H3K4me3 and H3K27me3 levels during seed germination. ChIP fractions were analyzed using PCR primers covering the promoter, UTR and gene body regions of *ABI3* (Figure 5A) or *DOG1* (Figure 5B). In wild-type *Arabidopsis* prior to seed germination (0 HAS), we detected relatively high levels of H3K4me3 at gene body chromatin regions but low levels at the promoter and 3′-UTR chromatin regions. H3K4me3 levels decreased drastically upon germination (24 HAS) and reached nearly background levels in 3-day-old seedlings (72 HAS). In contrast, H3K27me3 levels were very low at 0 HAS and 24 HAS but very high at 72 HAS. These data indicate that *ABI3* and *DOG1* repression is associated with the removal of the active transcriptional mark H3K4me3 and the establishment of the repressive transcriptional mark H3K27me3. Compared to Col-0, *al6 al7* and *Atbmi1a Atbmi1b* mutants showed overall higher levels of H3K4me3 and lower levels of H3K27me3 at the *ABI3* and *DOG1* chromatin (Figure 5), indicating a delay in the H3K4me3-to-H3K27me3 switch during seed germination and early seedling growth. A similar delay was also observed on *CRU3* and *CHO1* chromatin (Figure S5). Together these data indicate that *AL6/7* as well as *AtBMI1a/b* are necessary for the timely switch from H3K4me3-associated activation to H3K27me3-associated repression of seed developmental genes during germination and early seedling growth.

### AL6 and the PRC1 H3K27me3-reader component LHP1 bind chromatin of seed developmental genes

**Figure 6:**
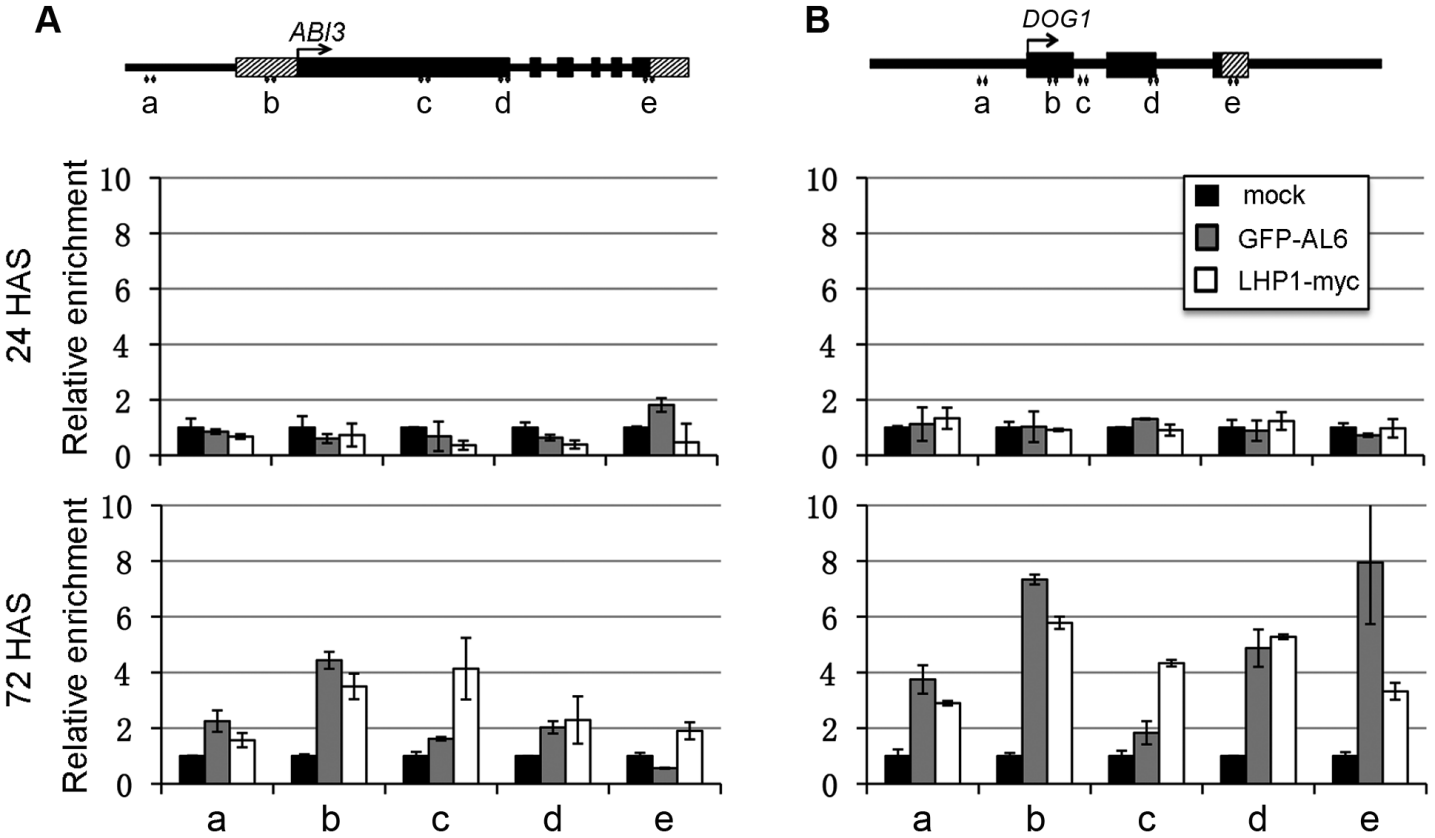
LHP1 and AL6 binding at *ABI3* and *DOG1* chromatin. Relative enrichments of LHP1-myc and GFP-AL6 proteins were analyzed at the five regions (a to e) of *ABI3* (A) and *DOG1* (B) loci. Transgenic seeds/seedlings expressing *LHP1-myc* or *GFP-AL6* were analyzed at 24 or 72 hours after stratification (HAS) by ChIP using anti-myc or anti-GFP antibodies. Samples in the absence of antibodies serve as negative controls (mock). Values were normalized to internal controls (relative to input and to *TUB2*). Data represent means ± SD of three biological replicates.

To examine whether AL6 binds directly to chromatin of seed developmental genes, we performed ChIP analysis using an anti-GFP antibody on the *pAL6:GFP-AL6* complemented *al6 al7* line. GFP-AL6 enrichment at *ABI3* (Figure 6A) and *DOG1* (Figure 6B) was undetectable in seeds at 0 or 24 HAS but was significantly elevated in seedlings at 72 HAS. Similarly, the H3K27me3-reader component of the PRC1 complex LHP1 [19] was observed to bind *ABI3* and *DOG1* chromatin in seedlings at 72 HAS (Figure 6), when using anti-myc antibody on the *pLHP1:LHP1-myc* complemented *lhp1* line [26]. These data indicate that AL6 and LHP1 directly bind chromatin of *ABI3* and *DOG1*. While the PHD-domains of AL1, AL4 and AL7 [24] as well as the full-length AL1, AL2, AL6 and AL7 proteins (Figure S6) bind H3K4me3 *in vitro*, we failed to detect GFP-AL6 binding of the *ABI3* and *DOG1* chromatin in seeds at 0 or 24 HAS when H3K4me3 levels are high. This inconsistency might be explained by failure to detect GFP-AL6 binding because of technical limitations associated with difficulties in ChIP analysis using seed material. Alternatively or additionally, the GFP-AL6 binding of H3K4me3-rich chromatin might be unstable and occur transiently prior to a more stable AL6-PRC1 chromatin association during *ABI3* and *DOG1* repression.

### A proposed model for the role of AL PHD-PRC1 in silencing switch of seed developmental genes during seed germination

**Figure 7:**
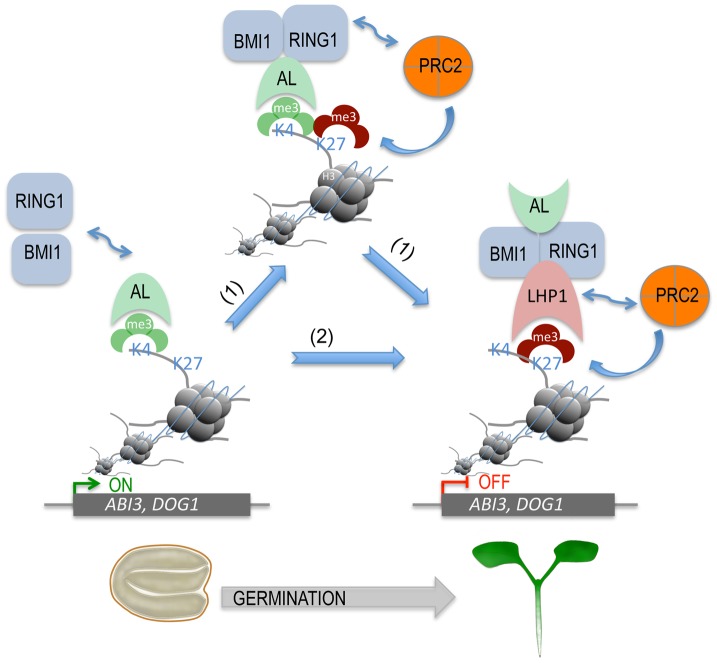
A proposed model for AL PHD-PRC1 complexes in silencing seed developmental genes during seed germination. ALs, *via* their highly conserved PHD domains, bind H3K4me3 of chromatin, triggering the recruitment of PRC1 components BMI1 and RING1 *via* AL-AtBMI1, AL-AtRING1, and AtBMI1-AtRING1 physical interactions. Next, two possible pathways (1 and 2) can lead to stable repressive chromatin state formation. In the first case (1), PRC2 is recruited *via* its subunit CLF interaction with AtRING1 and deposits H3K27me3, favoring further LHP1 recruitment *via* H3K27me3-LHP1 binding. In the second case (2), LHP1 is first recruited *via* its interaction with AtRING1 or AtBMI1, and then PRC2 is recruited *via* its subunit MSI1 interaction with LHP1 and deposits H3K27me3. In both cases, H3K27me3-LHP1 and PRC2 MSI1-LHP1 interactions form a positive loop in H3K27me3 enrichment. This hypothetic model can explain how seed developmental genes (*e.g. ABI3*, *DOG1*) are switched from active transcription to a stably repressed state, which is necessary for timely seed germination and proper seedling growth and development.

On the basis of our study, we propose a model for chromatin state switch of seed developmental gene silencing during seed germination (Figure 7). Seed developmental genes (e.g. *ABI3* and *DOG1*) are actively expressed in seeds and marked by the transcriptional activation chromatin marker H3K4me3. During germination, H3K4me3 provides a docking site for AL proteins (*e.g.* AL6, AL7, and possibly other ALs) *via* the conserved PHD domain recognition, which subsequently recruits the PRC1 ring-finger components RING1 (AtRING1A/B) and BMI1 (AtBMI1A/B) through physical protein-protein interactions. The recruitment of RING1 and BMI1 favours H3K27me3 deposition by PRC2 through two possible mechanisms. In the first case, RING1 directly recruits PRC2. In line with this, a physical interaction between AtRING1a and the PRC2 core component CURLY LEAF (CLF) was detected in a previous study [20]. Alternatively, PRC2 may be recruited through LHP1. In line with this, physical protein-protein interactions were observed between LHP1 and RING1 or BMI1 [20]–[22] and between LHP1 and the PRC2 component MULTICOPY SUPPRESSOR OF IRA 1 (MSI1) [27]. LHP1, *via* its chromodomain, binds H3K27me3 [28], [29], forming a positive feedback loop of PRC2-mediated H3K27me3 deposition and enhancing stable AL PHD-PRC1 complex formation. Stable repression of seed developmental genes guarantees timely seed germination and proper seedling growth. Beyond seed germination, the PRC1 components LHP1 and AtBMI1 had been reported as required for H3K27me3 deposition and repression of some target genes in roots and plants, likely involving other additional regulatory proteins [23], [27].

## Discussion

In *Arabidopsis*, the seven AL proteins together with the two ING (INhibitor of Growth) proteins form a family of small (235 to 270 aa in length) proteins containing a single PHD domain. The ING group proteins are conserved in plants, animals and fungi; the animal ING proteins bind H3K4me3 *via* their PHD domain and act as components of histone acetylase/deacetylase or chromatin-remodeling complexes involved in multiple critical processes (reviewed in Guérillon *et al.* [30]). The AL and ING proteins contain PHD domains of a similar primary sequence and tertiary structure, and consistently AL proteins also bind H3K4me3 ([24]; Figure S6). Distinctively, however, the ING proteins harbor at the N-terminus a conserved NCR (Novel Conserved Region) domain necessary for protein-protein interaction [30] whereas the AL proteins have a PAL domain, which is specific for plant proteins. Our study demonstrates that ALs could form dimers and could bind both AtBMI1 and AtRING1 (Figures 1 and 2). The AL6 N-terminus containing PAL is sufficient for binding with ALs and AtRING1a, indicating PAL as a novel protein-protein interaction domain. The finding of ALs as interactors of PRC1 components opens a new horizon for understanding the mechanistic function of this family of PHD-domain proteins.

While the function of the two *Arabidopsis ING* genes is unknown so far, our study has revealed a redundant function of *AL6* and *AL7* in the regulation of seed germination. The AL6 and AL7 proteins show the highest sequence homology among all AL proteins, and both *AL6* and *AL7* genes are expressed at high levels in seeds. While the *al6* and *al7* single mutants were normal, the *al6 al7* double mutant displayed a delay in germination under osmotic stress growth conditions (treatment with salt or mannitol; Figure 3). Similarly, the *Atbmi1a Atbmi1b* double mutant also displayed a germination delay. This is consistent with the proposed function of AL6/7 and AtBMI1a/b working in complexes to promote seed germination. The enhanced germination defects observed in the *al6 al7 Atbmi1a Atbmi1b* quadruple mutant indicate that AL6/7 and AtBMI1a/b may also work in parallel pathways and/or that other ALs as well as AtBMI1c may be involved in seed germination regulation. Furthermore, AL6/7 likely act earlier than AtBMI1a/b during seed germination. This is further evidenced by later growth phenotypes: the *Atbmi1a Atbmi1b* plants are characterized by the presence of ectopic embryonic traits [21], [22] whereas the *al6 al7* mutant plants are morphologically indistinguishable from Col-0. In line with the importance of AtRING1 within the PRC1 complex, the loss of both AtRING1a and AtRING1b caused ectopic embryonic callus formation in seedlings as well as many other growth and developmental defects [21]. Phenotype differences exhibited by the *lhp1* [31], *Atring1a Atring1b* [20], *Atbmi1a Atbmi1b* [21], [22] and *al6 al7* (this study) mutants also pinpoint to additional independent functions of *LHP1*, *RING1*, *BMI1* and *ALs* in diverse plant growth and developmental processes.

AL6/7 and AtBMI1a/b promote seed germination likely through repression of seed developmental genes. Consistently, the *al6 al7* mutant and the *Atbmi1a Atbmi1b* mutant showed derepression of *ABI3*, *DOG1*, *CRU1*, *CRU3*, *CHO1* and *PER1* (Figure 4). The expression of these seed developmental genes has been previously shown to negatively regulate seed germination [4], [7], [9], [10]. Quantitative differences in gene expression and the stress-inducible nature of these genes in seed germination regulation might explain the *al6 al7* mutant phenotype observable under osmotic stress conditions. The detected gene derepression was more severe and persisting in *Atbmi1a Atbmi1b* than in *al6 al7*. This indicates that although AL6/7 and AtBMI1a/b form protein complexes, they play distinct roles: AL6/7 acts early and transiently, whereas AtBMI1a/b are necessary for establishing stable repression. We propose that AL6/7 bind H3K4me3 and recruit AtBMI1a/b, facilitating H3K27me3 deposition and stable repressive AL PHD-PRC1 complex formation. Prior to seed coat rupture, all four analyzed target loci (*i.e. ABI3*, *DOG1*, *CRU3* and *CHO1*) were highly enriched in H3K4me3 and depleted of H3K27me3 (Figure 5), which is consistent with their high transcriptional activities. Upon seed germination, H3K4me3 levels decreased and H3K27me3 levels increased, leading to a repressive chromatin state characterized by background levels of H3K4me3 but high levels of H3K27me3. The switch from H3K4me3 to H3K27me3 is correlated with the seed germination time when assayed under varied physiological conditions [14]. Loss of AL6/7 or AtBMI1a/b caused a delay in the switch from H3K4me3 to H3K27me3, which is in agreement with the observed seed germination delay phenotypes. High levels of H3K27me3 at seed developmental genes were maintained by PRC2 throughout the subsequent seedling and vegetative growth stages [12], [13]. Consistently, binding of the PRC1 H3K27me3-reader component LHP1 was detected at *ABI3* and *DOG1* (Figure 6) as well as at *CRU3* and *CHO1* loci ([29]; http://epigenomics.mcdb.ucla.edu/cgi-bin/hgTracks).

It is worth noting that there is a marked correlation of gene conservation during evolution. ABI3 sequence homologues are found in evolutionarily distant species, including green algae, mosses, gymnosperms and angiosperms [32]. AL homologues also appeared together with green algae and are widespread in the green lineage. PRC1-related sequences have emerged later during green lineage evolution: BMI1 homologues are found as early as the mosses whereas RING1 and LHP1 homologues are found only in angiosperms [19]. This indicates that H3K4me3 and AL readers are established early whereas H3K27me3 and PRC1 readers appeared later in the evolution of seed plants. It will be of interest to investigate AL and BMI1 function in mosses to examine the hypothesis that the ABI3-regulatory pathway is ancient and acquired for desiccation tolerance beyond seed germination control [33], [34]. This will also examine minimum component composition required for AL PHD-PRC1 complex function.

Our finding of physical interactions between PHD-domain AL proteins and PRC1, two types of readers for the functionally opposite chromatin marks, H3K4me3 and H3K27me3, respectively, is novel and intriguing. In general, genome regions contain either the active mark H3K4me3 or the repressive mark H3K27me3. Bivalent configuration containing both H3K4me3 and H3K27me3 has been first reported in animal stem cells and is thought to maintain developmental genes in a silenced state poised for activation upon cell differentiation [35], [36]. A transient bivalent-like state with AL-H3K4me3 and PRC1-H3K27me3 interactions could exist at seed developmental genes during germination. Most remarkably, however, AL-H3K4me3 interaction likely serves to recruit PRC1 to previously active seed developmental genes, leading to the switch from on to off of transcription of these genes. In animals, the Polycomblike (PCL) proteins bind the active mark H3K36me3 *via* Tudor domain and physically interact with PRC2 to implement *de novo* repression of previously active embryonic stem cell-specific genes during transition to cell differentiation (reviewed in Abed and Jones [37]). The *Arabidopsis* PHD-domain proteins VRN5 and VIN3 form complexes with PRC2, which may act similarly to animal PCL-PRC2, in establishing repression of *FLOWERING LOCUS C* (*FLC*) for plant vegetative-to-reproductive growth phase transition [38]. Distinctively, our study proposes recruitment of PRC1 *via* AL-H3K4me3 interaction (Figure 7), which challenges the classic hierarchical paradigm where PRC2 is recruited prior to PRC1. In line with our proposition, two recent studies have shown that AtBMI1 positively regulates H3K27me3 enrichment at several genes including *ABI3* in 10-day-old plants [23] and LHP1 is required for H3K27me3 enrichment at flower gene loci in roots [27]. It is reasonable to speculate that different PRC1-associated factors may be involved in repression of different genes in different types of plant cells/tissues. Also an increasing number of studies in animals start to reveal varied composition of PRC1/PRC1-associated complexes as well as examples of H3K27me3 (PRC2)-independent functions of some PRC1 complexes [39], [40]. Future studies are necessary to investigate PRC1 composition and spatial-temporal dynamic complex assembly *in vivo*, which remain poorly documented so far.

In conclusion, our study demonstrates that the PRC1 core components AtBMI1 and AtRING1 physically interact with the PHD domain H3K4me3-binding proteins ALs and that the loss of AL6 and AL7 partly mimics the *Atbmi1a Atbmi1b* mutant phenotype in seed developmental gene derepression and seed germination delay. Our data supports a model in which the AL PHD-PRC1 complexes built around H3K4me3 lead to a switch from the H3K4me3-associated active to the H3K27me3-associated repressive transcription state of seed developmental genes. Reprogramming of gene activity is a mandatory step to allow plant growth phase transitions as well as cell differentiations in plants and animals. Our newly discovered mechanism may extend to other plant targets and be relevant to Polycomb silencing in other organisms. Our results also raise new questions. For instance, which enzymes are involved in the removal of H3K4me3? Whether the timely order of PRC1 and PRC2 recruitment is target context dependent? How is the target specificity determined? Answering these questions will undoubtedly shed further light on the molecular mechanisms of chromatin state switch, which is at the heart of gene reprogramming and cell differentiation.

## Materials and Methods

### Yeast two-hybrid assays

Full-length ORFs of *AL1*, *AL2*, *AL5*, *AL6* and *AL7* were PCR-amplified from *Arabidopsis* cDNA using gene specific primers (Table S1), cloned into pGEMT-easy (Promega) and then into pGBKT7 and/or pGADT7 vectors (Clontech, http://www.clontech.com) using *Bam*HI and *Xho*I restriction enzyme sites. pGBKT7-AtRING1a, pGBKT7-AtBMI1a and pGBKT7-LHP1 constructs have been described previously [20], [21]. The truncated constructs AtRING1aN (aa 1–218), AtRING1aM (aa 116–303), AtRING1aC (aa 238–522), and RING (aa 116–218) were obtained through PCR amplification and cloning. The truncated construct AL6N (aa 1–162) was recovered from the yeast-two-hybrid cDNA library screen using the bait plasmid pGBKT7-AtRINGA1a. The various combinations of pGBKT7- and pGADT7-based constructs were introduced into *Saccharomyces cerevisiae* strain PJ69-4a, selected on synthetic defined (SD) medium lacking Leu and Trp (SD-LT), and assayed for protein-protein interaction by growth on SD lacking Leu, Trp and Ade (SD-LTA). Details of constructs are described in *SI Materials and Methods*.

### Plant materials

All *Arabidopsis thaliana* lines were derived from the Columbia ecotype (Col-0). T-DNA insertion mutants were obtained for AL6 (SALK_040877) and AL7 (SALK_032503) from the *Arabidopsis* Biological Resource Center (ABRC, http://www.arabidopsis.org). The double mutant *al6 al7* was generated by crossing of the single mutants. The *Atbmi1a Atbmi1b* mutant [21] and transgenic *Arabidopsis* lines expressing *FLAG-AtRING1a* [20] or *LHP1-myc* [26] have been previously described. To generate the transgenic line expressing *GFP-AL6*, *AL6* cDNA was first introduced into the Gateway entry plasmid pENTR-3C and then by recombination into pB7WGF2 (http://gateway.psb.ugent.be/), resulting in the *GFP-AL6* fusion driven by the 35S promoter. Next, the *AL6* endogenous promoter sequence (1311 bp upstream of the start codon) was PCR-amplified and used to replace the 35S promoter in pB7WGF2:GFP-AL6 by cloning into *Hin*dII and *Spe*I restriction sites, resulting in pAL6:GFP-AL6. pAL6:GFP-AL6 plasmid was introduced into *Agrobacterium tumefaciens* (GV3101) and the resulting strain was used to transform *Arabidopsis*. Transgenic homozygous lines containing a single T-DNA insertion were obtained.

### GST pulldown assays

The *AL1*, *AL2*, *AL6* and *AL7* cDNAs were cloned into *Bam*HI and *Xho*I sites of pGEX-4T-1 expression vector. pGEX-4T-1-AtBMI1a and pGEX-4T-1-AtBMI1b constructs have been previously described [21]. All constructs were introduced into *E. coli* Rosetta (DE3) strain in which glutathione-S-transferase (GST) and GST-fusion proteins were expressed and purified. Total protein extracts from two-week-old *Arabidopsis* seedlings expressing *FLAG-AtRING1a* or *GFP-AL6* were used in pulldown assays performed as previously described [41]. The pulldown fractions were analyzed by Western blot using monoclonal antibodies against FLAG (Sigma Aldrich) or GFP (Miltenyi).

### Co-immunoprecipitation (Co-IP) assays

Two-week-old *Arabidopsis* seedlings expressing *GFP-AL6* and *FLAG-AtRING1a* or *FLAG-AtRING1a* alone were ground in liquid nitrogen and proteins were extracted in lysis buffer (50 mM Tris-HCl pH 7, 150 mM NaCl, 10% glycerol, 4 mM MgCl_2_, 0.5% Triton X-100, 1 mM DTT, anti-complete proteinase (Roche) and DNaseI (Fermentas)). The crude protein extract was filtered through Miracloth, cleared by centrifugation (20 mins, 10000 *g*) and subsequently pre-cleared for 1 hour with magnetic protein A beads (Magna-ChIP, Millipore). A fraction was conserved as input control. IP was carried out overnight at 4°C using polyclonal anti-GFP antibodies (Invitrogen) in combination with magnetic protein A beads. Beads were washed 3 times for 10 min in lysis buffer. Immunoprecipitated proteins were eluted by boiling, separated by 10% SDS-PAGE and detected by Western blotting using HRP conjugated anti-GFP (Miltenyi) or anti-FLAG (Sigma Aldrich) antibodies.

### Fluorescence lifetime imaging (FLIM) assays

The *AL1*, *AL2*, *AL6*, *AL7*, *AtRING1a*, *AtBMI1a*, *AtBMI1b* and *LHP1* cDNAs were PCR-amplified and introduced into the Gateway system and cloned as 3′ or 5′ in-frame fusions to RFP or GFP sequences in plant expression vectors downstream of the 35S promoter (pB7WGF2; pB7FWG2; pH7WGR2; pH7RWG2; http://gateway.psb.ugent.be/). Plasmids were introduced into *A. tumefaciens* (GV3101). Bacteria cultures grown overnight were centrifuged and pellets resuspended in 10 mM MgCl_2_ to an optical density of 0.5 at 600 nm and induced with 200 µM acetosyringone. Leaves of 4–5 week old *Nicotiana benthamiana* plants were coinfiltrated with an equimolar bacterial suspension of the two constructs to be tested. Confocal laser scanning images of protein co-localization and FLIM data were recorded 2 days post-infiltration (LSM-700, Carl Zeiss; LIFA frequency domain fluorescence lifetime imaging system, Lambert Instruments). The percentage of GFP fluorescence lifetime decay was calculated relative to the absence of RFP fusion protein as an average of 3 biological replicates, each recording over 30 nuclei. Proteins were considered to interact if the presence of RFP-tagged proteins decrease GFP fluorescence lifetime by more than 5%, a reference value established according to the negative control: RFP with GFP.

### Seed germination tests

Seeds were chlorine gas surface sterilized and sown on petri dishes containing the growth media: Murashige and Skoog (MS) salts, 0.8% agar with or without addition of 100 mM NaCl or 200 mM mannitol. To synchronize germination, seeds were stratified after sowing for 3 days at 4°C and subsequently transferred to a growth chamber (23°C, photoperiod 16 h light, 8 h dark). Germination rates were scored daily for 12 days following stratification. Seeds were considered to have germinated when radicle emergence was visible under a dissecting microscope.

### Quantitative RT-PCR analyses

RNA was extracted from *Arabidopsis* seeds/seedlings at 0, 24 and 72 HAS as previously described [42]. Reverse transcription was performed using Superscript III reverse transcriptase (Invitrogen). Relative levels of cDNA were quantified with SYBR-Green I master mix in the LightCycler 480-2 according to the manufacturer's instructions (Roche). RT-PCR primers were designed with the aid of the Universal ProbeLibrary Assay Design Center (Roche, http://www.roche-applied-science.com). All primer sequences are listed in Table S1. The efficiency of each primer pair was calculated by LinRegPCR [43]. cDNA levels were normalized to internal reference genes *At4g34270* and *At4g26410* which are transcriptionally stable during germination [44].

### Chromatin immunoprecipitation (ChIP) assays

ChIP was performed as previously described [45] with minor modifications required for adaptation of the protocol for seed tissue. Fixation time was extended to one hour and chromatin was pre-cleared with protein-A beads. Chromatin was immunoprecipitated overnight using antibodies against H3K4me3 (Millipore), H3K27me3 (Millipore), myc (Roche), GFP (Invitrogen) or without antibodies as negative control. Buffers described in Berr *et al.* [45] were supplemented with various detergents, *i.e.* 0.01% SDS and 0.1% Triton X-100 were added to low and high salt wash buffers and 1% sodium deoxycholate was added to LiCl wash buffer.

## Supporting Information

Figure S1 Amino acid sequence alignment of AL proteins. Sequences were identified by BLAST similarity searches of the AL6 PAL-domain in proteome databases of *Arabidopsis thaliana* (At), *Vitis vinifera* (Vv), *Medicago truncatula* (Mt), *Oriza sativa* (Os), *Brachypodium distachyon* (Bd) and *Volvox carteri* (Vc). Full-length protein sequences were aligned using the ClustalW2 software (http://www.ebi.ac.uk/Tools/msa/clustalw2/). Residues with above 90% occurrence are coloured. Note that all sequences are characterized by an N-terminal PAL-domain (green box) and C-terminal PHD-domain (red box).
(JPG)

Figure S2 Phylogenetic tree analysis of AL proteins. The ClustalW2 aligned sequences were adjusted in Jalview. Phylogenetic reconstruction was performed using MEGA5.05 with the maximum likelihood as statistical method. The confidence of the clustering was evaluated by the bootstrap method using 200 replications. The plant species abbreviations are similar as in Figure S1.
(JPG)

Figure S3 Expression of *ALs* and seed developmental genes during seed germination. Relative expression levels of the indicated seed developmental genes (A, B) and *AL* genes (C) were analyzed by quantitative RT-PCR using seeds/seedlings grown on MS or MS supplemented with 100 mM NaCl (MS+NaCl) at 0, 24 and 72 hours after stratification. Relative expression levels are indicated on a LOG scale. Data represent means ± SD of three biological replicates.
(TIF)

Figure S4 Expression of *ALs* after seed germination and during different plant developmental stages. (A, B) Epifluorescent (left panel) and bright-field differential interference contrast (right panel) images of a seedling at 72 hours after stratification from the *pAL6:GFP-AL6* complemented *al6 al7* line and the wild-type Col-0, respectively. Note the green fluorescence specifically detected for GFP-AL6. (C) Expression levels of *ALs* shown as absolute signal intensity of microarray analysis. The aquaporin *TIP4;1* (*Tonoplast Intrinsic Protein 4;1*) serves as a reference gene. The data are retrieved from the AtGenExpress database (http://www.weigelworld.org/resources).
(JPG)

Figure S5 Relative levels of H3K4me3 and H3K27me3 in the *CRU3* and *CHO1* chromatin during seed germination in Col-0, *al6 al7* and *Atbmi1a Atbmi1b*. H3K4me3 and H3K27me3 levels were analyzed by ChIP at two regions (a, b) of *CRU3* (A) and *CHO1* (B). Gene structures are schematically represented by black boxes for exons, black lines for introns and promoters, and dashed boxes for untranslated regions. Seeds/seedlings at 0, 24 and 72 hours after stratification (HAS) were analyzed. Values were normalized to internal controls (relative to input and to *TUB2*). Data represent means ± SD of three biological replicates.
(TIF)

Figure S6 AL1, AL2, AL6 and AL7 bind H3K4me3. Agarose beads coated with GST, GST-AL1, GST-AL2, GST-AL6 or GST-AL7 were incubated with an equal aliquot of commercially purchased calf-thymus histones (Sigma). Pulled-down proteins were probed with anti-H3K4me3 antibody (upper panel). Coomassie blue stained membrane serves as loading control (bottom panel). Positions of GST and GST-tagged AL proteins are indicated by stars.
(TIF)

Table S1 Sequences of primers used in this study.
(XLSX)
