# Supplementary figures and images for: Arabidopsis AL PHD-PRC1 Complexes Promote Seed Germination through H3K4me3-to-H3K27me3 Chromatin State Switch in Repression of Seed Developmental Genes
Source: PLoS Genet. 2014 Jan 23;10(1):e1004091. doi: 10.1371/journal.pgen.1004091 (PMC3900384; doi:10.1371/journal.pgen.1004091)

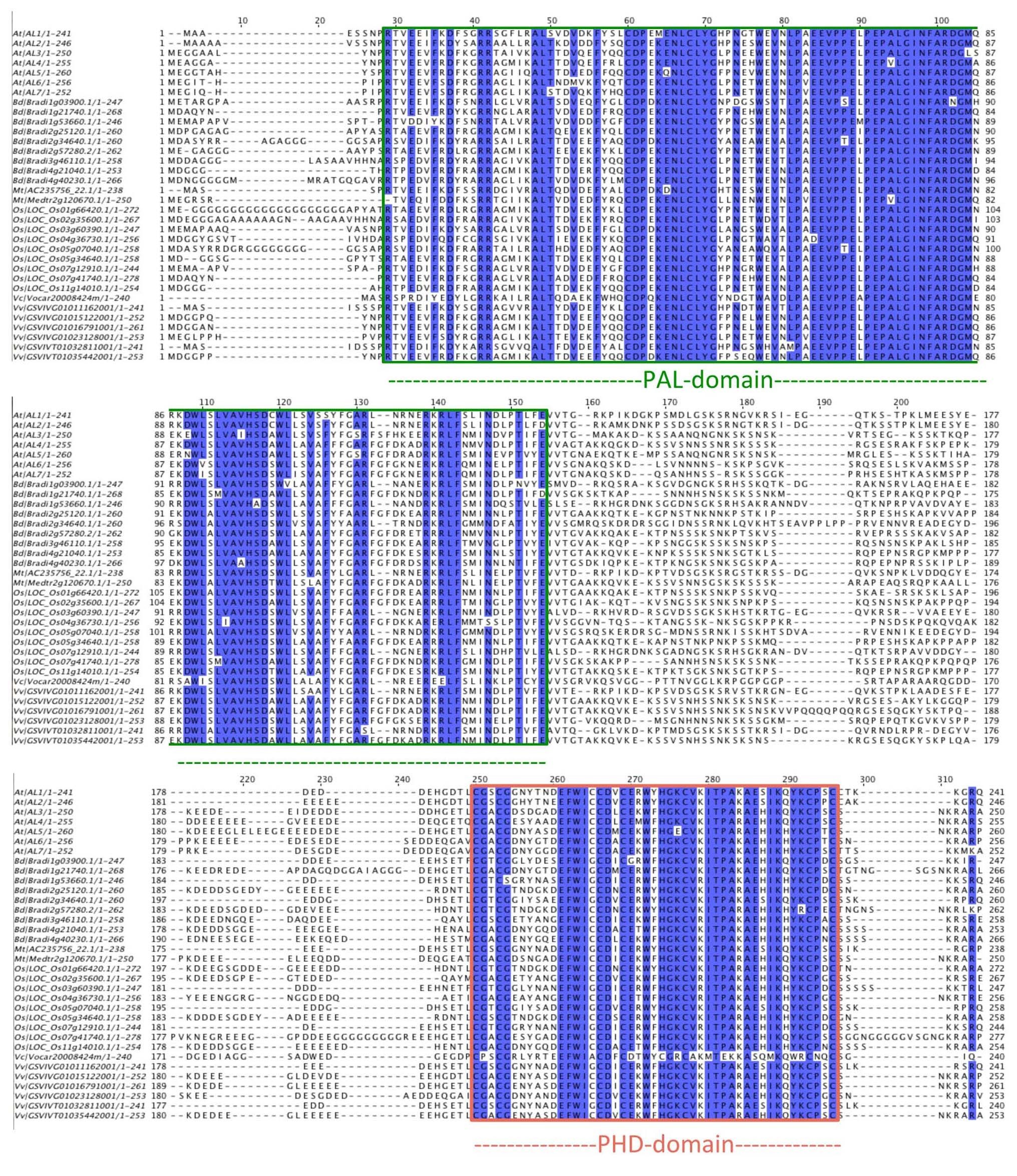

Supplement: Figure S1 — Amino acid sequence alignment of AL proteins. Sequences were identified by BLAST similarity searches of the AL6 PAL-domain in proteome databases of Arabidopsis thaliana (At), Vitis vinifera (Vv), Medicago truncatula (Mt), Oriza sativa (Os), Brachypodium distachyon (Bd) and Volvox carteri (Vc). Full-length protein sequences were aligned using the ClustalW2 software (http://www.ebi.ac.uk/Tools/msa/clustalw2/). Residues with above 90% occurrence are coloured. Note that all sequences are characterized by an N-terminal PAL-domain (green box) and C-terminal PHD-domain (red box). (JPG) [file pgen.1004091.s001.jpg]

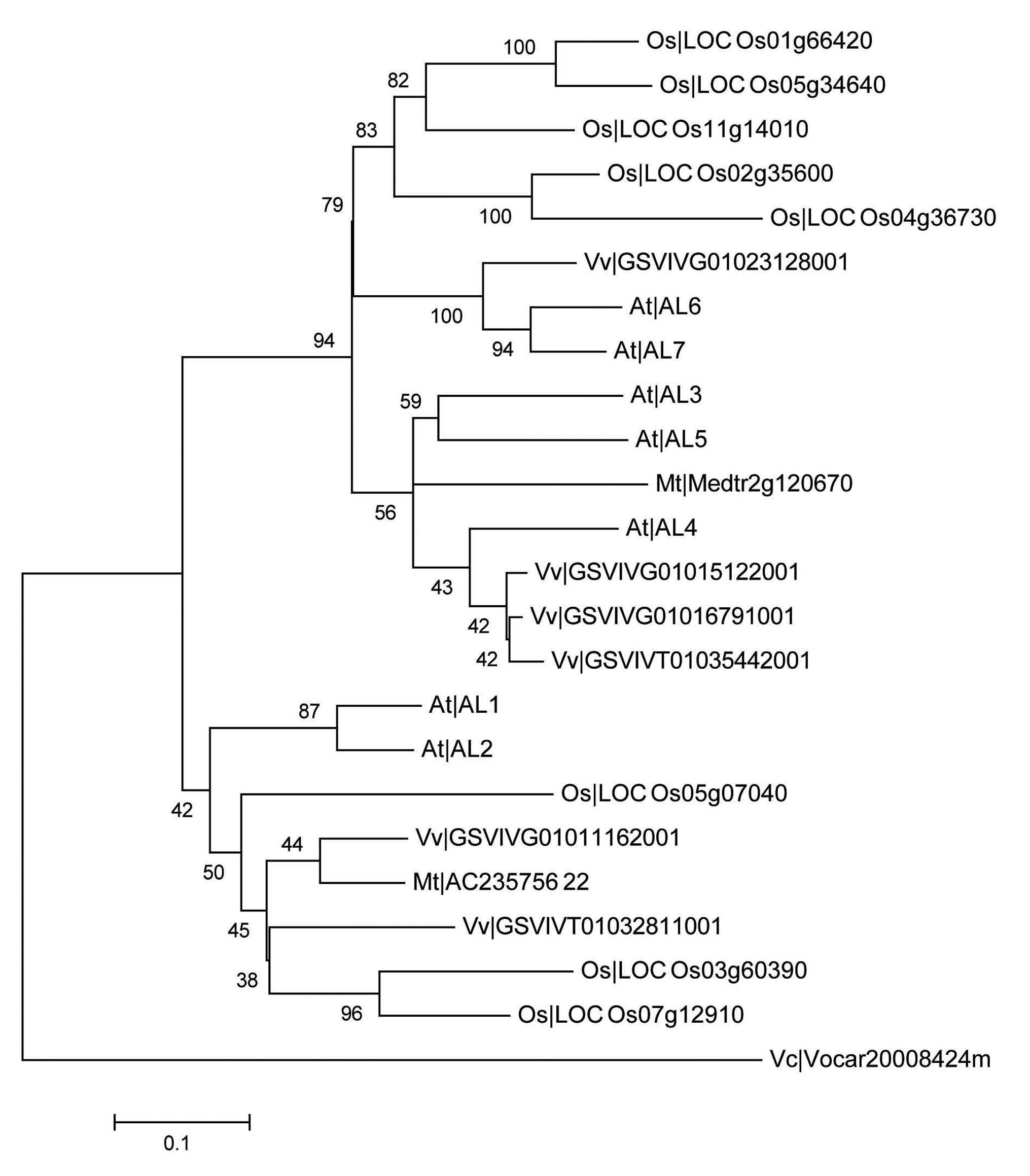

Supplement: Figure S2 — Phylogenetic tree analysis of AL proteins. The ClustalW2 aligned sequences were adjusted in Jalview. Phylogenetic reconstruction was performed using MEGA5.05 with the maximum likelihood as statistical method. The confidence of the clustering was evaluated by the bootstrap method using 200 replications. The plant species abbreviations are similar as in Figure S1. (JPG) [file pgen.1004091.s002.jpg]

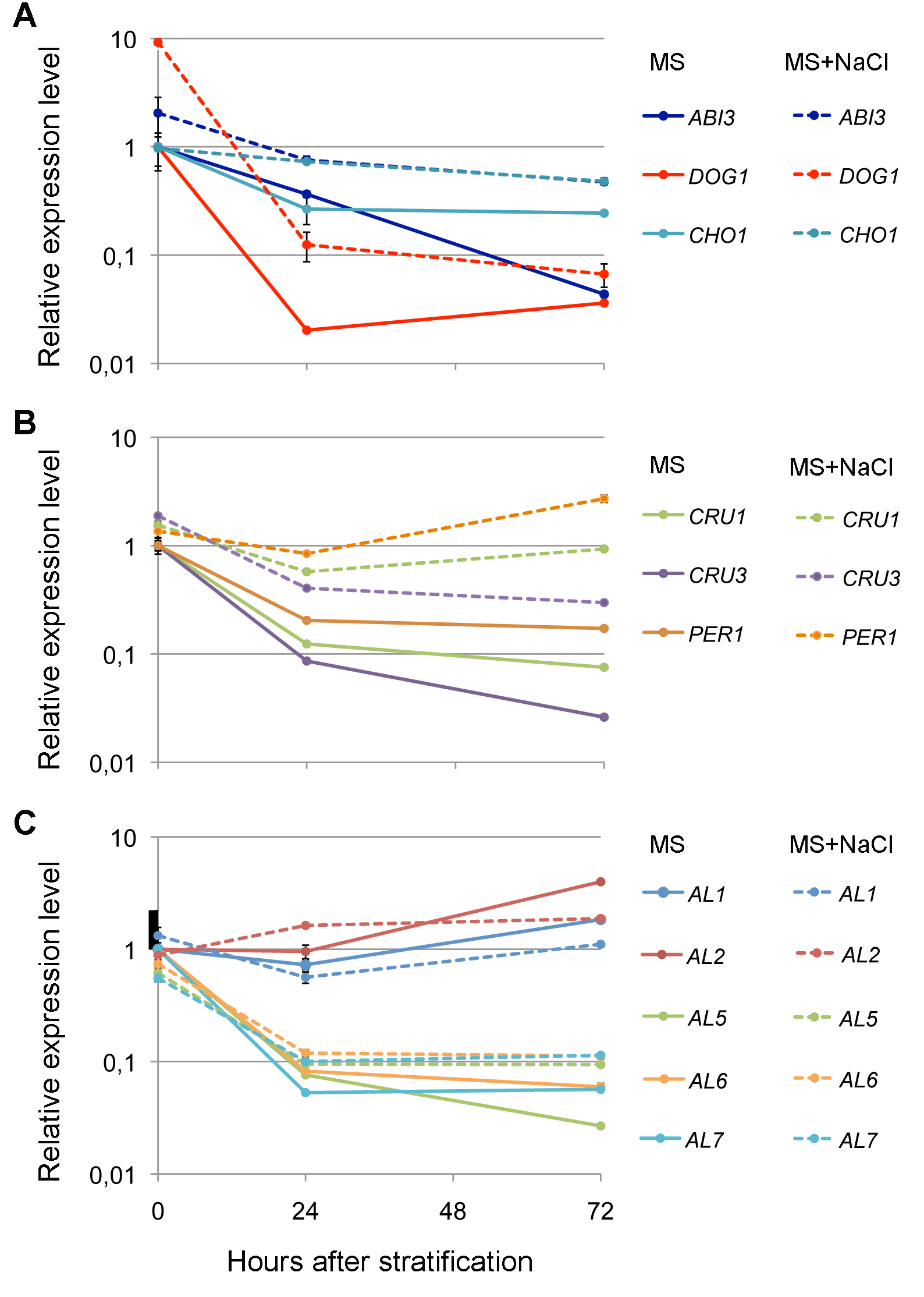

Supplement: Figure S3 — Expression of ALs and seed developmental genes during seed germination. Relative expression levels of the indicated seed developmental genes (A, B) and AL genes (C) were analyzed by quantitative RT-PCR using seeds/seedlings grown on MS or MS supplemented with 100 mM NaCl (MS+NaCl) at 0, 24 and 72 hours after stratification. Relative expression levels are indicated on a LOG scale. Data represent means ± SD of three biological replicates. (TIF) [file pgen.1004091.s003.tif]

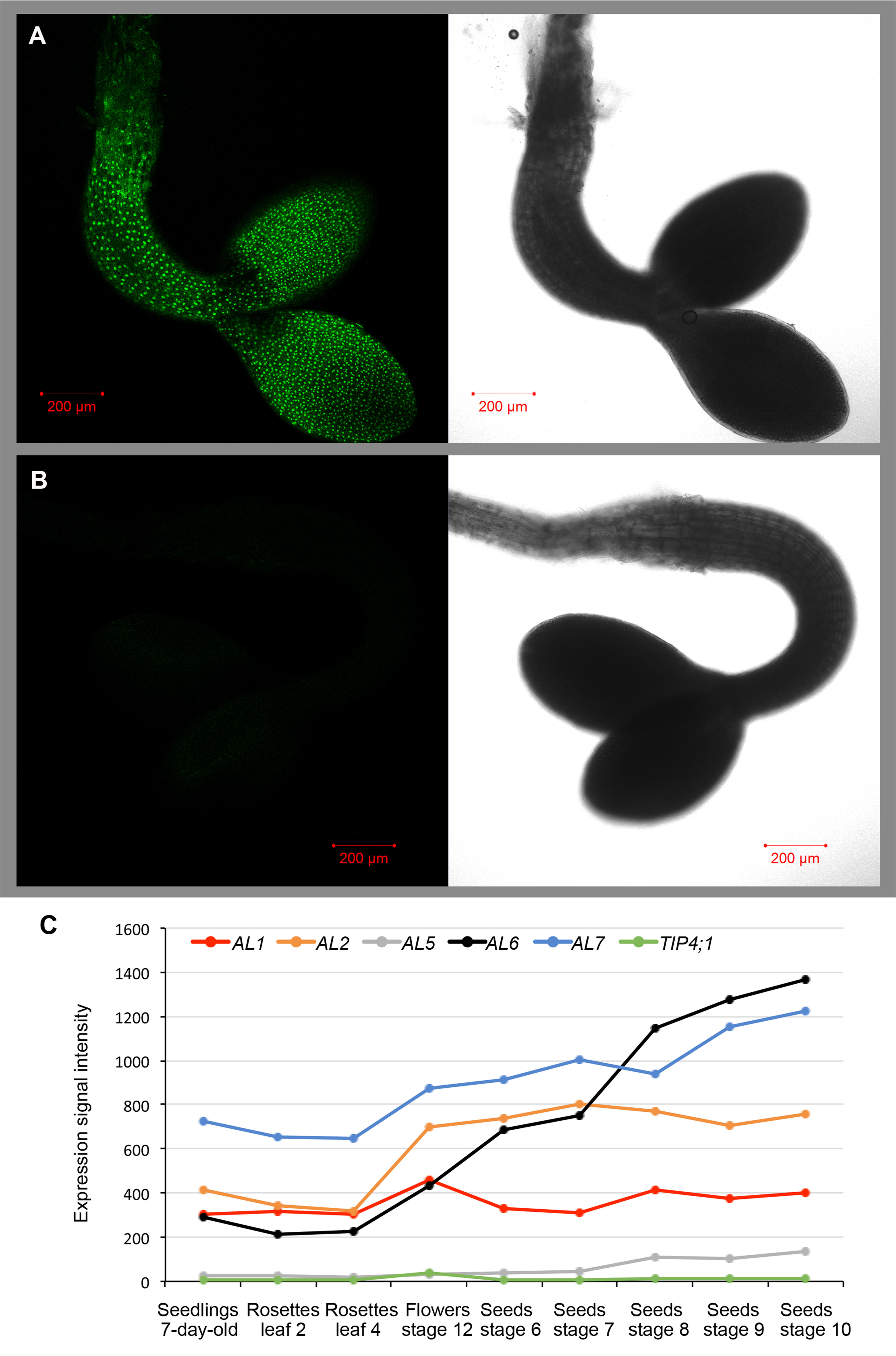

Supplement: Figure S4 — Expression of ALs after seed germination and during different plant developmental stages. (A, B) Epifluorescent (left panel) and bright-field differential interference contrast (right panel) images of a seedling at 72 hours after stratification from the pAL6:GFP-AL6 complemented al6 al7 line and the wild-type Col-0, respectively. Note the green fluorescence specifically detected for GFP-AL6. (C) Expression levels of ALs shown as absolute signal intensity of microarray analysis. The aquaporin TIP4;1 (Tonoplast Intrinsic Protein 4;1) serves as a reference gene. The data are retrieved from the AtGenExpress database (http://www.weigelworld.org/resources). (JPG) [file pgen.1004091.s004.jpg]

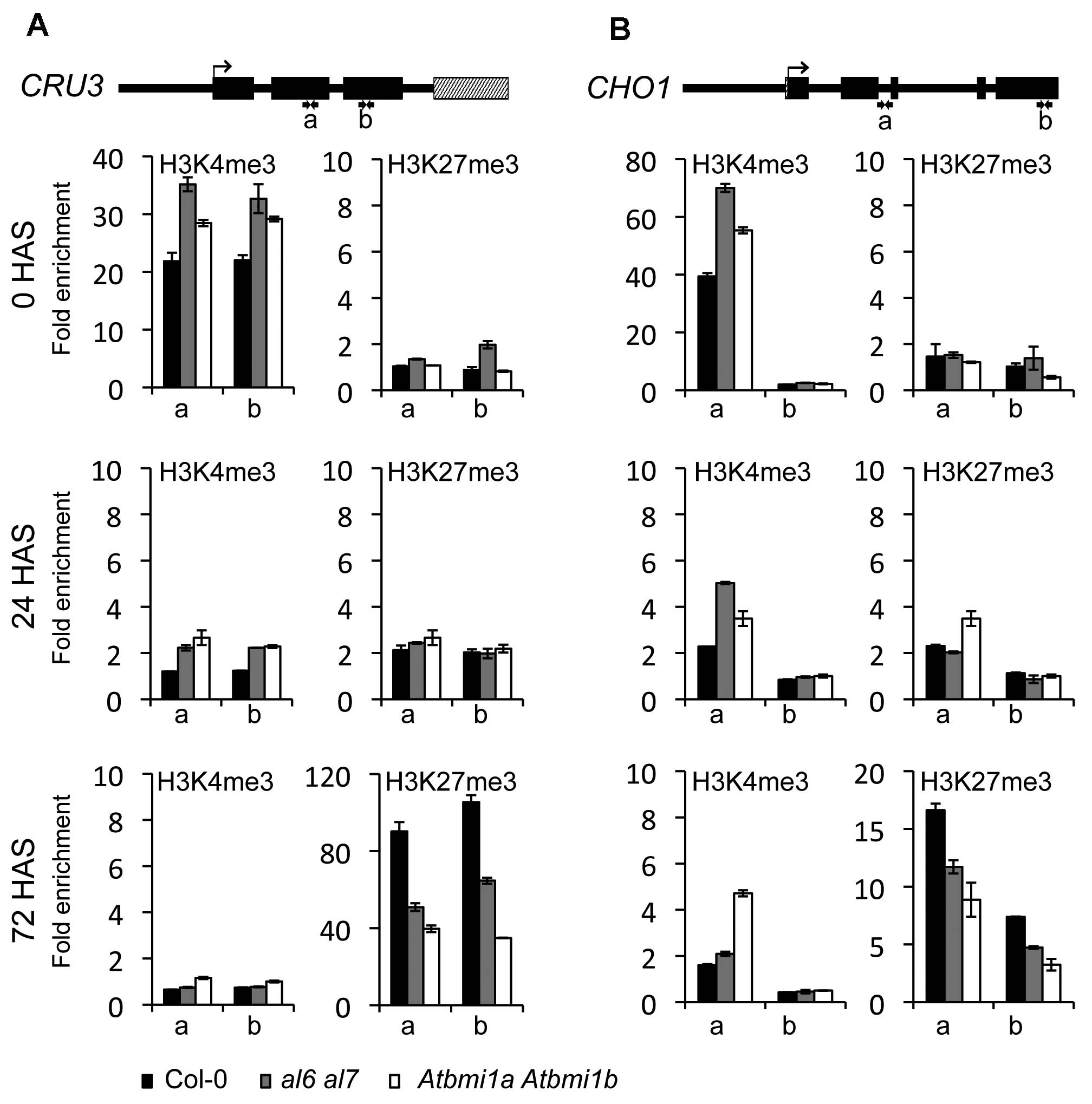

Supplement: Figure S5 — Relative levels of H3K4me3 and H3K27me3 in the CRU3 and CHO1 chromatin during seed germination in Col-0, al6 al7 and Atbmi1a Atbmi1b. H3K4me3 and H3K27me3 levels were analyzed by ChIP at two regions (a, b) of CRU3 (A) and CHO1 (B). Gene structures are schematically represented by black boxes for exons, black lines for introns and promoters, and dashed boxes for untranslated regions. Seeds/seedlings at 0, 24 and 72 hours after stratification (HAS) were analyzed. Values were normalized to internal controls (relative to input and to TUB2). Data represent means ± SD of three biological replicates. (TIF) [file pgen.1004091.s005.tif]

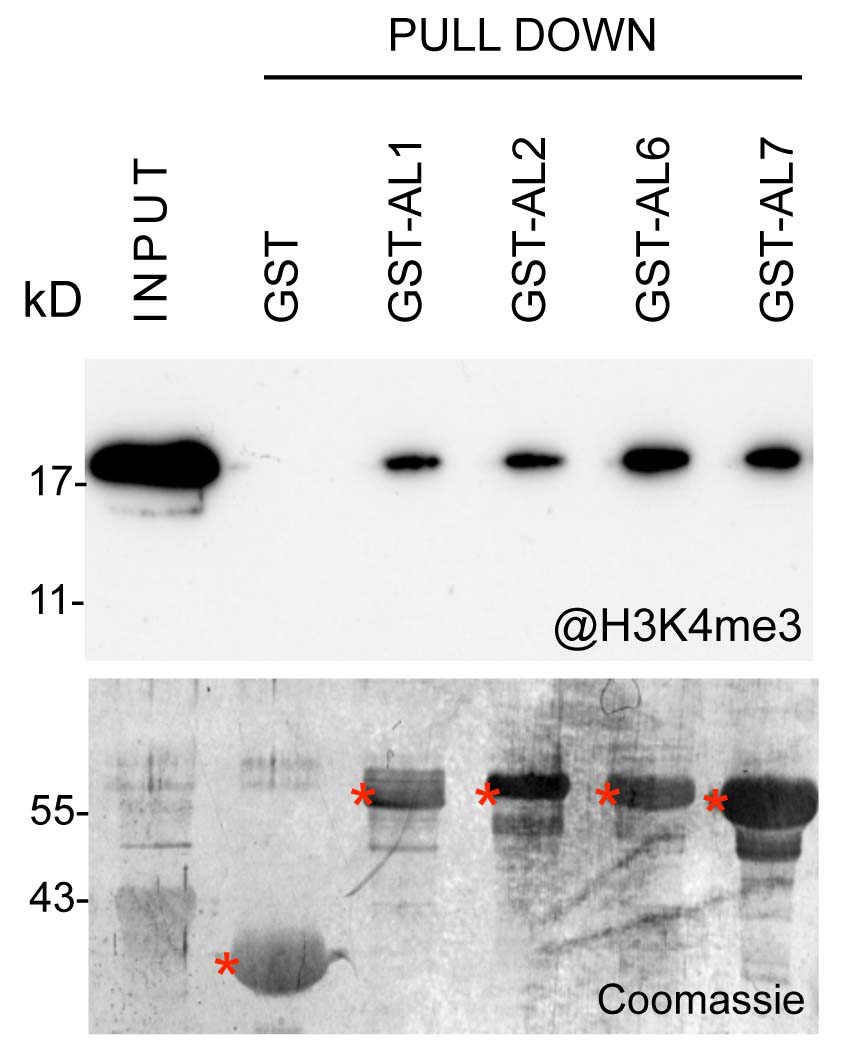

Supplement: Figure S6 — AL1, AL2, AL6 and AL7 bind H3K4me3. Agarose beads coated with GST, GST-AL1, GST-AL2, GST-AL6 or GST-AL7 were incubated with an equal aliquot of commercially purchased calf-thymus histones (Sigma). Pulled-down proteins were probed with anti-H3K4me3 antibody (upper panel). Coomassie blue stained membrane serves as loading control (bottom panel). Positions of GST and GST-tagged AL proteins are indicated by stars. (TIF) [file pgen.1004091.s006.tif]
